# Fluorescent Alloyed CdZnSeS/ZnS Nanosensor for Doxorubicin Detection

**DOI:** 10.3390/bios13060596

**Published:** 2023-05-31

**Authors:** Svetlana A. Mescheryakova, Ivan S. Matlakhov, Pavel D. Strokin, Daniil D. Drozd, Irina Yu. Goryacheva, Olga A. Goryacheva

**Affiliations:** Department of General and Inorganic Chemistry, Chemistry Institute, Saratov State University Named after N.G. Chernyshevsky, Astrakhanskaya 83, 410012 Saratov, Russia; meshcheryakova.s.a@gmail.com (S.A.M.); goryachevaiy@mail.ru (I.Y.G.)

**Keywords:** quantum dots, doxorubicin, nanosensor, fluorescence quenching, Stern–Volmer constants, anthracycline antibiotics, human plasma

## Abstract

Doxorubicin (DOX) is widely used in chemotherapy as an anti-tumor drug. However, DOX is highly cardio-, neuro- and cytotoxic. For this reason, the continuous monitoring of DOX concentrations in biofluids and tissues is important. Most methods for the determination of DOX concentrations are complex and costly, and are designed to determine pure DOX. The purpose of this work is to demonstrate the capabilities of analytical nanosensors based on the quenching of the fluorescence of alloyed CdZnSeS/ZnS quantum dots (QDs) for operative DOX detection. To maximize the nanosensor quenching efficiency, the spectral features of QDs and DOX were carefully studied, and the complex nature of QD fluorescence quenching in the presence of DOX was shown. Using optimized conditions, turn-off fluorescence nanosensors for direct DOX determination in undiluted human plasma were developed. A DOX concentration of 0.5 µM in plasma was reflected in a decrease in the fluorescence intensity of QDs, stabilized with thioglycolic and 3-mercaptopropionic acids, for 5.8 and 4.4 %, respectively. The calculated Limit of Detection values were 0.08 and 0.03 μg/mL using QDs, stabilized with thioglycolic and 3-mercaptopropionic acids, respectively.

## 1. Introduction

**Figure 1:**
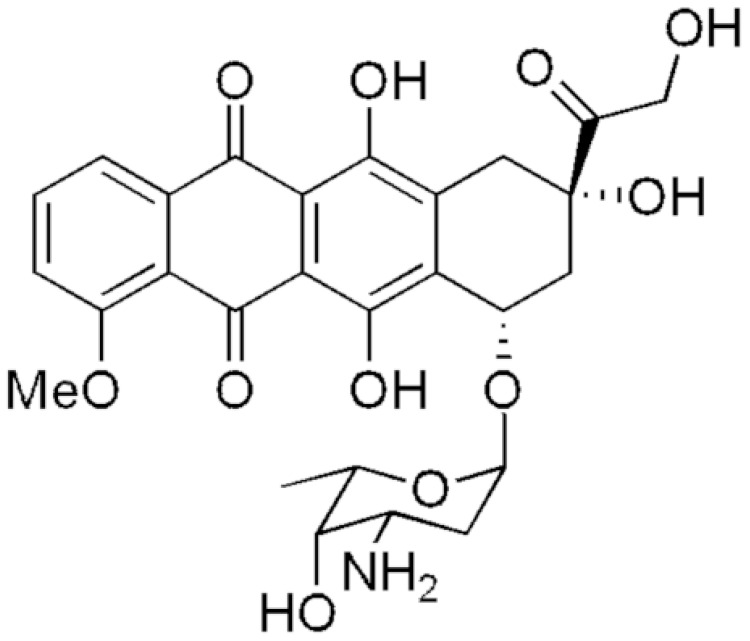
Doxorubicin structure.

Doxorubicin (DOX) is an effective chemotherapeutic agent from the group of anthracycline antibiotics (Figure 1), and has high activity against many types of cancer. It is one of the commonly used agents in the treatment of different cancers types, including pediatric cancer, leukemia, breast cancer, etc. [1]. However, the use of DOX has significant disadvantages because it affects both cancer and healthy cells. DOX is highly cardiotoxic, cytotoxic and neurotoxic. It causes cell death through multiple intracellular targets: DNA-adduct formation, topoisomerase II enzyme inhibition, reactive oxygen species generation, histone eviction, Ca^2+^ and iron hemostasis regulation and ceramide overproduction [2]. There are several mechanisms of DOX-induced cardiotoxicity, among which oxidative stress, free radical generation and apoptosis are the most widely reported [1]. The side effects are often observed when the concentration of DOX exceeds a certain level in blood, so the therapeutic range of DOX concentration in blood is narrow. For this reason, the continuous monitoring of DOX concentrations in body fluids is important [3].

Number methods are known for quantitative DOX determination in body fluids and tissues. One way is to measure the DOX concentration by registering its own absorption in the visible spectral range. In this case, a thorough purification of the sample or extraction is necessary. Such sample preparation takes up to one hour and allows for DOX determination with a Limit of Detection (LOD) of ~1.2 µg/mL DOX in plasma [3]. Spectrophotometric detectors are also used in tandem with high performance liquid chromatography and provide low DOX LOD values of 5–5000 ng/mL in rat serum and tissues. Here, performing a sample extraction using a methanol-chloroform mixture is also necessary. The method is able to determine the DOX concentration in both blood and tissues [4,5], as well as in gall and lymph [6]. The optical properties of DOX also allow its concentration to be determined on the basis of its emission. Thus, the DOX passage across the blood–brain barrier in the form of a low-density lipoprotein receptor-targeted liposomal drug was studied by Pinzón-Daza et al. DOX concentrations can be determined based on its fluorescence, at excitation and emission wavelengths of 475 and 553 nm, respectively [7]. DOX concentrations as low as 18 ng/mL in rabbit plasma were detected by capillary electrophoresis with in-column double optical-fiber LED-induced fluorescence detection using rhodamine B as an internal standard. Before analysis, rabbit serum samples were subsequently diluted twice with acetonitrile to precipitate the proteins. For electrophoretic separation, a borate buffer (15 mM, pH 9.0) containing 50% acetonitrile (*v*/*v*) was used [8]. Electrochemical methods are traditionally used for operative high-throughput detection. Differential pulse cathodic stripping voltammetry on a polished silver solid amalgam electrode in a specially designed micro-volume voltametric cell was used for the DOX determination with a LOD of 0.44 μM. The applicability of this method was verified through an analysis of spiked tap water samples and human urine [9]. The same authors developed differential pulse voltammetry on a polarized liquid/liquid interface impregnated with a ionic liquid polyvinylidenfluoride microporous filter with a DOX LOD of 0.84 μM. Due to the affection of some body fluids interfering compounds, this method must be preceded by a separation step. The ability of DOX to bind with DNA was used in a voltammetric DNA sensor using a glassy carbon electrode covered with electropolymerized Azure B film and physically adsorbed native DNA. In optimal conditions, the DNA sensor provided a DOX LOD of 0.07 nM for commercial DOX formulations and on artificial samples mimicking the electrolyte content of human serum [10]. The modification of the glassy carbon electrode with a vertically-ordered mesoporous silica-nanochannel film with electrochemically reduced graphene oxide by a one-step electrochemically assisted self-assembly method allowed us to reach a DOX LOD of 0.77 nM in human whole blood [11]. The application of rolling circle amplification allowed us to develop an ultrasensitive electrochemical DNA sensor with DOX@tetrahedron-Au as the electrochemical indicator, and reach a DOX LOD of 0.29 fM [12]. The application of surface enhanced raman spectroscopy for DOX detection using silica nanoparticles covered with 10 nm think gold film allowed us to archive a LOD of 20 nM in undiluted serum [13]. These methods for determining DOX concentrations are complex and costly and/or need exhaustive sample preparation.

DOX has a fairly intense native fluorescence in the range of 540–660 nm (quantum yield, QY is 4.39%) [14]. However, the optical properties of DOX (absorption and emission) are sensitive to the form of DOX molecules in the solution, which in turn, depends on plenty of factors, including DOX concentration, ionic strength, pH, additives and so on [15]. DOX, like other anthracyclines, has the propensity to form dimers and associates; DOX fluorescence dramatically drops upon dimerization (QY~10^−5^) [16]. This makes trouble for direct fluorescence-based DOX detection. There is a demand for the development of simple nanosensors that enable the fast determination of analytes in solutions based on changes in the properties of nanoparticles as they interact with analytes [17,18]. One example of such a system is localized surface plasmon resonance technology (LSPR). This LSPR-based biosensing system utilizes the sensitivity of the plasmonic frequency to changes in the local index of refraction at the nanoparticle surface. Optimizing the nanoparticle material and geometry alters the plasmonic properties towards sensitivity improvement [19,20].

An important property of DOX is the effective quenching of the emission of a large number of luminophores, which allows for the use of quenching-based methods for DOX detection. Different luminophores were used as emission turn-off probes, such as fluorescent polyethyleneimine-functionalized carbon dots [21] and other carbon nanostructures [22,23], gold nanoclusters [24], and phosphorescent Mn-doped ZnS quantum dots (QDs) [25], fluorescent Mn-doped ZnSe D-dots [26] and CdSe/ZnS QDs [27]. The DOX quenching of nanoparticles fluorescence can be so efficacious that the DOX quenching of QDs emission has been used for the detection of DNA [26] and analysis of telomerase activity [27]. While characterizing the DOX—luminophore interaction, the authors mostly focus on the quenching in model solutions, demonstrating perspectives for its application in biofluids.

In this work, we use fluorescent alloyed semiconductor QDs, which are of particular interest because they are photostable, homogeneous in size and properties and can form a stable aqueous colloid. In addition, the possibility to select surface ligands to achieve optimal interactions and improve the sensitivity of turn-off nanosensors is important and useful [28,29]. The analytical application of the quenching of QD fluorescence is based on changes in the emission intensity during the interaction between the nanosensors and modulating molecules (quenchers). The quenching of QD probe fluorescence is the simplest analytical method for DOX detection compared to other known protocols. Compared with the carbon-based nanostructures mentioned above, semiconductor QDs have excellent uniformity, high synthesis reproducibility, photo- and chemical stability, as well as well-studied synthetic routs and emission mechanism. As QDs, we used alloyed CdZnSeS/ZnS core/shell nanocrystals, covered with 3-mercaptopropionic acid (MPA) and thioglycolic acid (TGA) (Figure S1 in Supplementary Material). Compared to traditional core/shell QDs, alloyed QDs are characterized by a simple synthesis route and smaller size, while maintaining narrow emission peaks and high emission QY, and the possibility to use various surface ligands. The comparison of two QD samples with the same architecture of a semiconductor core, but stabilized with different surface ligands, makes it possible to determine the effect of the ligand on the sensitivity of QDs to emission quenching.

## 2. Materials and Methods

### 2.1. Materials

Cadmium (II) acetate (99.995%), zinc acetate (99.99%), zinc stearate (technical grade), elemental selenium (powder), elemental sulfur (powder), trioctylphosphine (90%), 1-octadecene (90%), oleic acid (90%), MPA and TGA were purchased from Merck. All other chemicals and solvents were of analytical grade and used without additional purification. In this work, we used Sindroxocin (50 mg, Actavis, Iceland), an antitumor antibiotic whose active ingredient is doxorubicin hydrochloride (50 mg). It also contains the following excipients: methyl parahydroxybenzoate (5 mg) and lactose monohydrate (263.15 mg). Before analysis, 3 mg (corresponding to 0.5 mg of DOX) of the lyophilizate was dissolved in 1 mL of Milli-Q water. From the resulting solution (500 µg/mL), solutions with concentrations of 50, 5 and 1 µg/mL were obtained.

### 2.2. QD Synthesis and Hydrophilization

The alloyed QD synthesis was adapted from [30]. For hydrophilization, 25 µmol of TGA or MPA were added to 50 µL of QDs aliquot in 1 mL of toluene. The following steps were performed according to [30].

### 2.3. QD Characterization and Spectral Measurements

Absorption spectra were obtained with a Shimadzu UV-1800 spectrophotometer (Shimadzu, Kyoto, Japan). Emission spectra were recorded with a Cary Eclipse fluorescence spectrometer (Agilent Technologies, Santa Clara, CA, USA) and multimode microplate reader Synergy H1 (BioTek Instruments, Charlotte, NC, USA). The ζ-potential of the QDs samples were determined via dynamic light scattering measurements using a Zetasizer Ultra instrument (Malvern Instruments, Malvern, UK). A Libra 120 transmission electron microscope (TEM) (Carl Zeiss, Jena, Germany) was used to take photomicrographs and determine the size of QDs.

### 2.4. Quantum Yield Calculation

The relative QY of QD emission was calculated as described in [31], relative to fluorescent dye Coumarin 153 (ethanol solution, peak optical density 0.1, QY 53%). The excitation and emission wavelengths of the QY measurement were 360 and 514 nm, respectively.

$$\Phi_{x}=\Phi_{st}\frac{I_{x}}{I_{st}}\frac{A_{st}}{A_{x}}\frac{n_{x}^{2}}{n_{st}^{2}}$$

Φ*_x_*—relative QY of the QDs sample;
Φ*_st_*—the relative QY of the reference (coumarin-153);
*I_x_*—is the integral fluorescence intensity of the QDs sample;
*I_st_*—integral intensity of the reference (coumarin-153);
*A_x_*—optical density of the QDs sample;
*A_st_*—optical density of the reference (coumarin-153);
*n_x_*—the refractive index of the sample (water);
*n_st_*—the refractive index of the reference solvent (ethyl alcohol).

### 2.5. Optical Measurements

In order to avoid the internal filter effect and maintain the same QD concentration in the samples, all QD samples were brought to the same optical density equal to 0.1 at excitation wavelength (λ_ex_ = 360 nm). All experiments were performed in three repetitions to evaluate the accuracy of the measurements.

### 2.6. Analysis Performance in Plasma Samples

Syndroxocin solution in Milli-Q (1000 mg/mL) water was used to determine the DOX concentration in plasma. It was added to plasma to obtain DOX concentrations in the range of 0.5, 1, 5, 10, 50, 100 and 500 μg/mL. Plasma was incubated for 30 min at 37 °C. The test was performed in a 96-well plate, and 50 µL of QDs colloid with an optical density of 0.1 was added to each well. Subsequently, 50 µL of spiked plasma was added to the QDs colloid and measured immediately. To evaluate the stability of the quenching magnitude, the incubation of DOX with plasma samples for 24 h was also performed.

## 3. Results and Discussion

### 3.1. Optical Properties of QDs

The nanosensor for DOX detection is based on QD fluorescence quenching. The most probable quenching mechanisms are the Förster resonance energy transfer (FRET) and photoinduced electron transfer (PIET). Therefore, to enhance quenching, it was necessary to obtain QDs with a fluorescence peak overlapping with the absorption band of DOX (Figure S2) [32,33].

**Figure 2:**
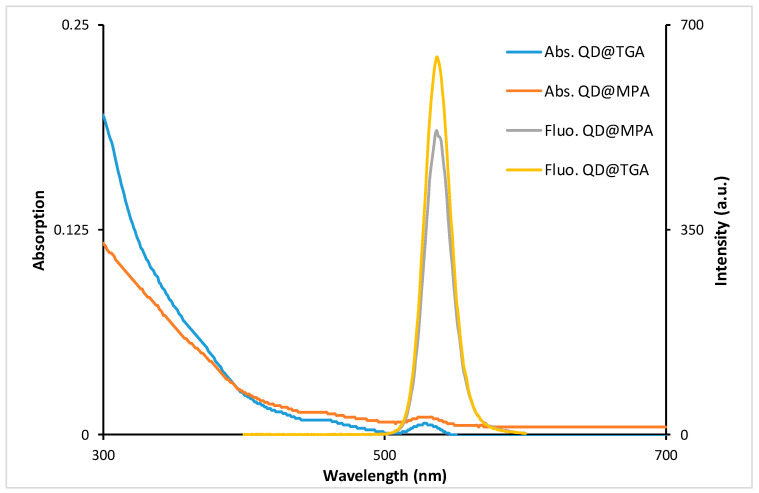
Absorbance and fluorescence (λ_ex_ = 360 nm) spectra of QDs stabilized with 3-mercaptopropionic acid (QD@MPA) and thioglycolic acid (QD@TGA).

CdZnSeS/ZnS QDs (λ_em_ = 540 nm) (Figure 2) were synthesized and transferred into water by the ligand exchange process according to the procedure described by Drozd et al. [30]. TEM images show a size of about 10 nm (Figure S3). As hydrophilizing agents, two mercaptoacids with a different carbon chain length—TGA (two carbon atoms) and MPA (three carbon atoms)—were used (Figure S1). After TGA coverage, the zeta potential of QD is—76 mV, and for MPA it was—46 mV. The emission QY for QD@TGA and QD@MPA was calculated as 0.55 and 0.60, respectively.

It is important to note that in most cases, studies were performed with pure DOX hydrochloride, while chemotherapy often uses DOX formulations with excipients, which can affect the efficacy and accuracy of assays. In this work, we used the pharmaceutical formulation Syndroxocin, which contains Doxorubicin hydrochloride (50 mg), methyl p-hydroxybenzoate (1 mg) and lactose monohydrate (50 mg). To control the possible effect of lactose on QDs emission, we added lactose (5 mg/mL) to the QD solutions and compared the optical properties. The results show that no changes in the QD fluorescence intensity and spectra shape were observed, so we can conclude that lactose has no effect on QDs fluorescence (Figure S4).

### 3.2. Optical Properties of DOX

DOX has a characteristic wide absorption peak at 450–550 nm (λ_max_ = 480 nm) and fluorescence spectra with three distinct peaks at 560, 594 and 638 nm. DOX molecules in solutions can be presented in different forms, which are reflected in their spectral properties.

**Figure 3:**
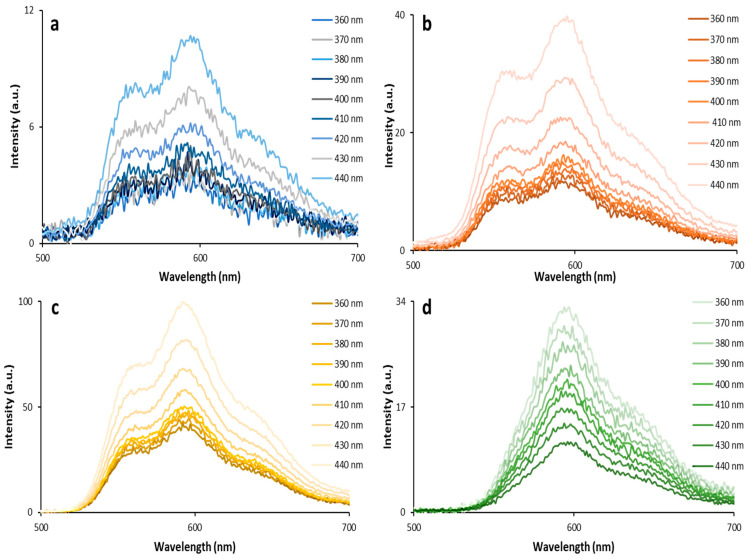
3D fluorescence spectra of DOX aqua solutions with concentrations of 1 µg/mL (**a**), 5 µg/mL (**b**), 50 µg/mL (**c**) and 500 µg/mL (**d**).

As can be seen from the 3D fluorescence spectra (Figure 3), there is no shift in the DOX emission maxima when excited with the light of different wavelengths and when the DOX concentration is changed. At the same time, there are notable changes in the ratio of peaks when the DOX concentration increases. These effects are related to the dimerization of DOX as well as the inner filter effect. Thus, DOX undergoes dimerization with increasing concentration. An increase in the absorption intensity at 415–540 nm indicates the formation of aggregates [16].

### 3.3. Influence of QDs on the DOX Fluorescence

Various possible mechanisms of the interaction between QDs in the excited state and DOX molecules have been suggested, including FRET and PIET. Since the emission spectra of QDs and the absorption spectra of DOX overlap (Figure S2), we assume that FRET is the main reason for QD emission quenching. Because both QDs and DOX are emissive, the mutual influence of QDs and DOX molecules on each other’s fluorescence should be checked.

**Figure 4:**
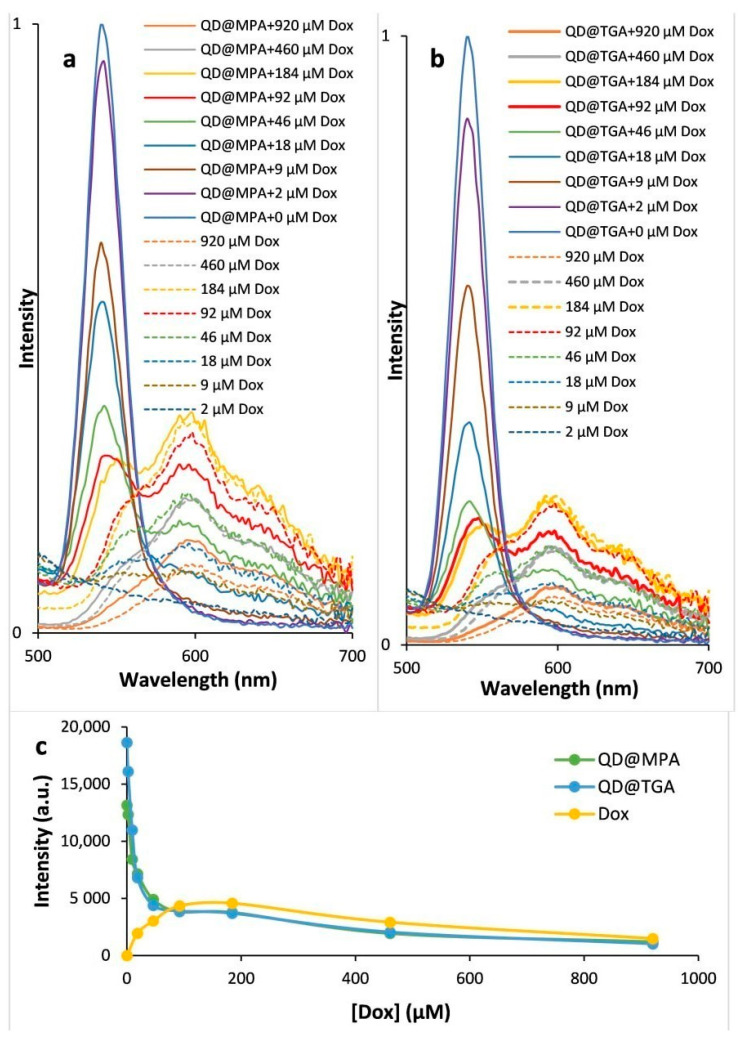
Normalized fluorescence spectra (λ_ex_ = 360 nm) of QDs stabilized by MPA (**a**) and TGA (**b**) in the presence of different DOX concentrations (solid lines), for comparison DOX fluorescence spectra are presented (dashed lines); (**c**) intensity of fluorescence of DOX, QD@MPA + DOX and QD@TGA + DOX solutions (λ_ex_ = 360 nm, λ_em_ = 540 nm), depending on DOX concentration.

To examine the possible effect of QDs on DOX, fluorescence spectra were recorded for a series of DOX and QD-DOX solutions with an increasing concentration of DOX and a fixed concentration of QDs (Figure 4). It was shown that the fluorescence intensity of DOX concentrations < 50 μM is lower than the fluorescence intensity of QDs at a peak near 540 nm. This suggests that at DOX concentrations around or above 50 μM, the contribution of DOX to the total fluorescence intensity of QD-DOX solutions will be very significant. A DOX concentration < 18 μM practically does not contribute to the total QD-DOX fluorescence.

At the same time, when QDs are added to the DOX solution, the fluorescence spectra of DOX are almost unchanged. However, at high concentrations (>50 μM), a slight quenching of the fluorescence in the presence of the QDs occurs, which may be due to the inner filter effect. This effect is clearly seen from the comparison of the intensities of the fluorescence of the DOX, QD@MPA + DOX and QD@TGA + DOX solutions at increasing DOX concentrations (Figure 4c). The shape of the DOX spectrum has not visibly changed, indicating that there are no changes in the molecule.

### 3.4. QDs Fluorescence Quenching by DOX

**Figure 5:**
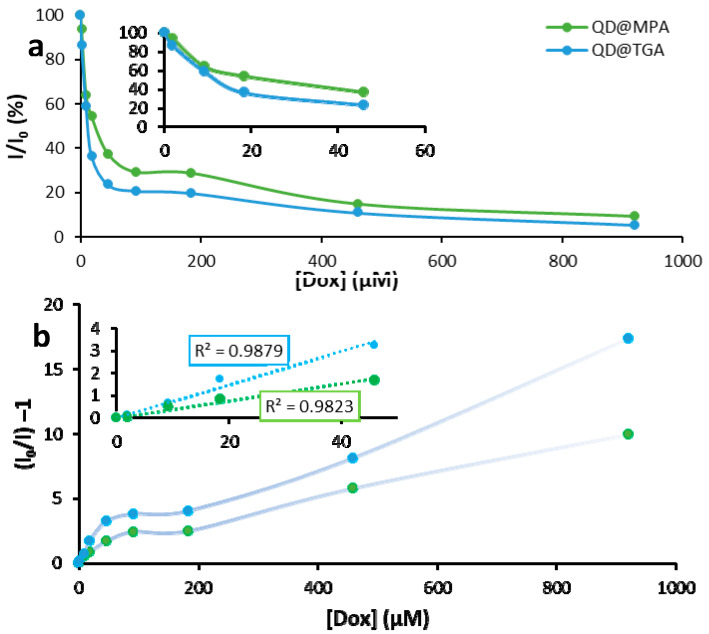
Dependence of normalized QDs emission on DOX concentration (**a**) and Stern–Volmer plots (**b**); insert (**b**): calculation of Stern–Volmer constants for 0–50 μM DOX.

Since the DOX concentration used for chemotherapy is large (244 mg per square meter) [34], we studied a wide range of DOX concentrations. To determine the magnitude of QDs fluorescence quenching with DOX, 0–920 μM (0 to 500 μg/mL) DOX concentrations (Table S1) at fixed QDs concentration were used. The comparison was made for QDs coated with TGA and MPA to find the more sensitive QDs (Figure S1). Since quenching is based on the adsorption of DOX on the QD surface, the modifying surface layer can play a key role. The obtained dependences of QDs emission intensity from DOX concentrations are presented in Figure 5a,b. It is possible to see two different ranges; at low DOX concentrations (<50 μM), there is the quenching of QDs emission, and at the range > 100 μM of DOX, an approximately stable emission intensity is observed due to the increasing contribution of DOX fluorescence. For the evaluation of the quenching of QDs fluorescence, Stern–Volmer plots were built and Stern–Volmer constants were evaluated (Formula (1)).

(1) $$\frac{I_{0}}{I}=1+k_{SV}[DOX]$$

*I*_0_ and *I*—fluorescence intensity in the absence and presence of DOX, [DOX]—concentration of the DOX [mol/L], *k_SV_*—Stern–Volmer constant [L/mol]. The deviation of the Stern–Volmer plot from liner dependency suggests that several processes accompany quenching and that the nature of quenching may combine both static and dynamic interactions (Figure 5b) [35].

The plots are linear for a DOX concentration of 0–50 μM, which corresponds to the range in which there is no significant DOX fluorescence effect on the total emission of QD-DOX solutions. The value of the Stern–Volmer constants was calculated at 0.074 M^−1^ for QD@TGA and 0.039 M^−1^ for QD@MPA. The quenching for QD@TGA in the presence of DOX is about two times more effective than that for QD@MPA. Two reasons, both related to the surface ligand size, can cause such a difference. The smaller size of the TGA molecules provides a better interaction of the quenching agent on the QD surface with the emissive semiconductor core. On the other hand, the smaller size of the TGA molecule makes the hydrophilic ligand layer on the QD surface more dynamic, opening up more opportunities for DOX interactions with the QD surface.

### 3.5. Determination of DOX in Human Blood Plasma

**Figure 6:**
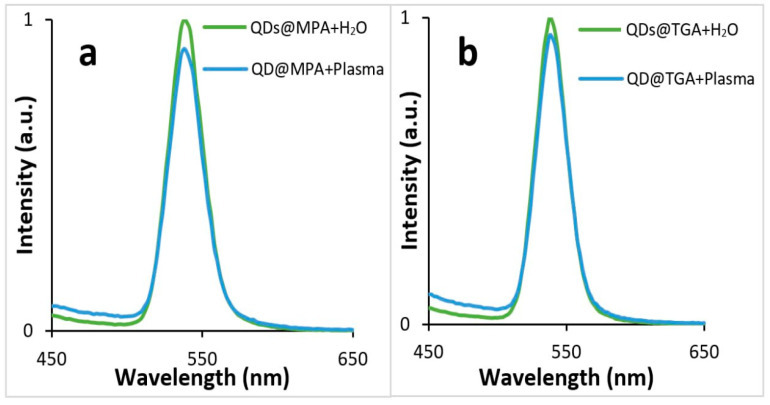
Fluorescence of QDs in water and plasma: TGA stabilized QDs (QD@TGA) (**a**) MPA stabilized QDs (QD@MPA) (**b**).

DOX is usually determined in patients’ blood, plasma or urine. The intrinsic feature of DOX as an anthracycline antibiotic is its ability to bind with DNA and other compounds. The determination of DOX concentration can be inaccurate due to its elimination from the sample with biomaterials. Therefore, for DOX determination, it is important to use biofluid samples that are as unaltered as possible. To determine the feasibility of analysis in biological fluids, the matrix effect of plasma on QDs fluorescence was tested. For this purpose, 50 μL of plasma was added to 50 μL of the QDs colloid in the well of a microtiter plate. As a result, a slight change in the fluorescence intensity can be seen due to the inner filter effect of the serum components and the change in the pH in comparison with the previous experiment, which was performed in Milli-Q water (Figure 6) [36].

**Figure 7:**
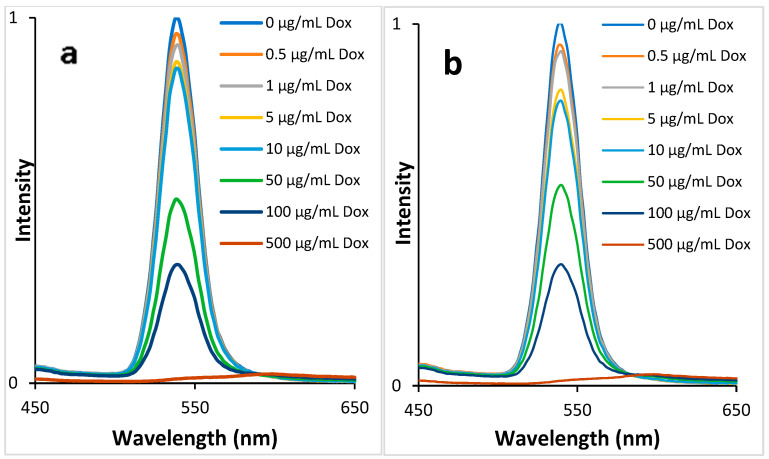
Normalized fluorescence spectra of QDs stabilized with MPA (**a**) and TGA (**b**) in the presence of plasma spiked with different DOX concentrations.

Freshly prepared Syndroxocin solutions were used for the spiking plasma experiments. Incubation at body temperature and stirring were carried out in order to uniformly distribute DOX over the plasma components and obtain an equilibrium of possible interactions. The Corning Costar 96-well plate was chosen as the plate with the least adsorbing effect for bio-objects. The 96-well plate also allows you to build a calibration curve and use the test for multiple samples at once. The fluorescence spectra show that the concentration of DOX in the plasma affects the QDs fluorescence. Moreover, the fluorescence intensity of TGA-stabilized QDs decreases more than that of MPA-stabilized QDs as well as DOX in water solutions (Figure 7). Consequently, TGA-stabilized QDs are more sensitive to DOX in plasma, but their dependence on concentration is not sufficiently uniform throughout the DOX concentration range. Nevertheless, QD@MPA shows a more uniform dependence of fluorescence quenching on DOX concentration, which may be due to the increased stability of QD@MPA relative to QD@TGA over a wide range of pH [37].

**Figure 8:**
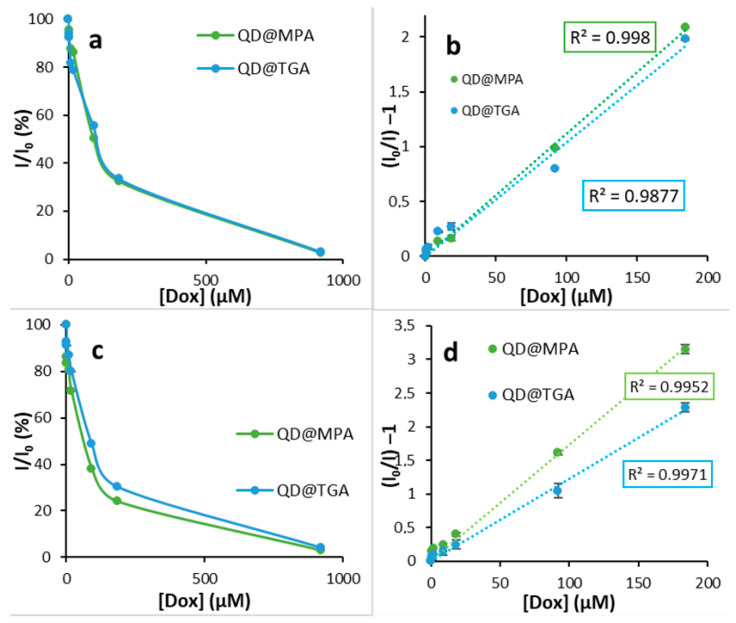
Dependence of QDs fluorescence intensity of spiked plasma vs. DOX concentration (**a**,**c**) and Stern–Vomer plots (**b**,**d**) after 30 min (**a**,**b**) and 24 hours (**c**,**d**) incubation.

To reveal the dependence of the QDs fluorescence intensity decrease on the DOX concentration, we plotted the fluorescence quenching profiles and Stern–Volmer plots (Figure 8). The DOX concentration range was chosen taking into account DOX dosing recommendations. The linear range of DOX concentrations was 0–184 μM (corresponding to 0–100 μg/mL), with a Stern–Volmer constant of 0.011 M^−1^ for QD@TGA and 0.010 M^−1^ for QD@MPA, so that the fluorescence reduction values could be determined accurately. The better linearity of Stern–Volmer plots in plasma (R^2^ = 0.977 and 0.9809 for 0–184 μM of DOX) compared to Stern–Volmer plots in aqua solutions (R^2^ = 0.9634 and 0.9765 for a more narrow DOX concentration range of 0–50 μM) may result from the increased stability of QDs coatings with TGA and MPA in more alkaline media, and thus the uniform interaction of DOX with the QD surface [38]. A DOX concentration of 0.5 µM in plasma was reflected in a decrease in the fluorescence intensity of QDs, stabilized with thioglycolic and 3-mercaptopropionic acids, for 5.8 and 4.4%, respectively. To calculate the Limit of Detection (LOD) values, two approaches were used. The calculated instrumental LOD value by Formula (S1) was 4.0 ng/mL for QDs@TGA and 1.2 ng/mL for QDs@MPA, because of the low deviation in the QDs emission intensity measurements. So, we used an approach based on the standard deviation of response in the presence of DOX (Formula (S2)), and obtained more realistic DOX LOD values of 0.06 μg/mL for QDs@TGA and 0.02 μg/mL for QDs@MPA.

Since plasma has been found to affect QDs fluorescence quenching, we decided to try diluting the plasma by a factor of 10 when preparing the samples. The results showed that the dilution resulted in a significant change in QDs sensitivity due to a dramatic decrease in the DOX concentration. Therefore, undiluted plasma was used for further studies.

Because the half-life of DOX in the human body varies between 30 and 150 h [39], DOX is prescribed in several cycles. Each cycle consists of several days with an injection of DOX until therapeutic blood concentrations are achieved [40]. Thus, there is a period of DOX accumulation until the next injection in one cycle. Therefore, in order to simulate a round in the intrinsic cycle, we analyzed the spiked plasma with different concentrations of DOX plasma samples after incubation for 24 h. QDs fluorescence quenching in the presence of DOX was preserved even after this long incubation period (Figure 8), indicating the high affinity of DOX with the QDs surface and stability of the obtained equilibrium between the DOX and plasma components. The Stern–Volmer plot has liner dependence in the area of 0–184 μM. The Stern–Volmer constants were 0.017 M^−1^ for QDs@TGA and 0.012 M^−1^ for QDs@MPA.

Due to the fact that after 24 h, the Stern–Volmer constant values experienced minor changes, but the plots’ linearity was better, we assume that both static and dynamic interactions may have played a contribution. The LOD values for DOX detection after 24 hours incubation in plasma were calculated by Formula (S2), and it was 0.08 μg/mL for QDs@TGA and 0.03 μg/mL for QDs@MPA.

## 4. Conclusions

The ability of DOX to quench the fluorescence of alloyed CdZnSeS/ZnS QDs was used to develop turn-off fluorescence nanosensors for DOX determination in human blood plasma. The quenching depends on the concentration of DOX in the solution. By comparing the magnitude of quenching for two samples of QD covered with mercaptoacids (TGA and MPA), the effect of surface hydrofilic ligands was shown:

The QDs covered with shorter carbon chain TGA are more sensitive to DOX presence both in aqua solution and in plasma. The smaller ligand predetermines slightly less reproducibility of QD fluorescence intensity values. With longer carbon chain MPA, the dependence of fluorescence on the DOX concentration has smaller coefficients of variation. The quenching system was also implemented in blood plasma. In this case, the blood sample preparation only consists of obtaining plasma and does not need any additional purification. It is interesting to mention our unexpected discovery that the magnitude of QD fluorescence quenching is higher in plasma than in aqua, resulting in about a double increase in Stern–Volmer constants, as well as additionally increasing the plasma long incubation with DOX. The quenching of QDs fluorescence in the DOX-spiked plasma has a linear dependence in the range of 0–200 µM.

DOX calculated LOD values for fluorescence turn-off nanosensors were 0.08 μg/mL for QDs@TGA and 0.03 μg/mL for QDs@MPA, respectively (we used 24 h DOX incubation with plasma before assay). The suggested format using undiluted plasma without any sample preparation and lengthy complex analysis methods opens the way for routine studies of DOX transformation in the patient’s body and the selection of individual chemotherapy protocols. An easy-to-use nanosensor has been developed and could become a meaningful tool for medical applications, as it can be used as an indicator for chemotherapy protocol correction.

The developed nanosensor can be used to monitor the concentration of DOX in patients through cycles of chemotherapy in order to optimize the cytostatic dose. Potentially, the developed nanosensor (after appropriate studies) can be used for the detection of other anthracycline cytostatics, as they are able to bind to the surface of the nanosensor and quench its emission. The issue of selectivity is not a limiting factor for nanosensors in biofluids, since the chemotherapy regimen and the particular drugs are known for each patient. The lack of selectivity for specific anthracycline antibiotics can be a problem, for example, in the analysis of hospital wastewater. In this case, the properties of the nanosensor must be optimized for each specific task.

## Supplementary Materials

The following supporting information can be downloaded at: https://www.mdpi.com/article/10.3390/bios13060596/s1, Figure S1: Formulas of (a) Thioglycolic acid and (b) 3-mercaptopropionic acid; Figure S2: An overlap between the QDs fluorescence spectrum (red) and DOX absorption spectrum (blue); Figure S3: Fluorescence spectra of QDs stabilized with 3-mercaptopropionic acid (A) and thioglycolic acid (B) with and without lactose; Figure S4: Fluorescence spectra of QDs stabilized with 3-mercaptopropionic acid (a) and thioglycolic acid (b) in X10 diluted plasma, spiked with DOX; Table S1: Correspondence of DOX concentration in µg/mL and µM (DOX Molecular Weight 543.5).
