# Supplementary material for: Fluorescent Alloyed CdZnSeS/ZnS Nanosensor for Doxorubicin Detection
Source: Biosensors (Basel). 2023 May 31;13(6):596. doi: 10.3390/bios13060596 (PMC10296636; doi:10.3390/bios13060596)
Supplement: Supplementary file 1 [file biosensors-13-00596-s001.zip › biosensors-2365207-supplementary.pdf]

biosensors

MDPI

Supplementary Material

Fluorescent Alloyed CdZnSeS/ZnS Nanosensor for Doxorubicin Detection

Svetlana A. Mescheryakova, Ivan S. Matlakhov, Pavel D. Strokin, Daniil D. Drozd, Irina Yu. Goryacheva and Olga A. Goryacheva *

Saratov State University named after N.G. Chernyshevsky, Department of General and Inorganic Chemistry, Chemistry Institute, Astrakhanskaya 83, 410012 Saratov, Russia; meshcheryakova.s.a@gmail.com (S.A.M.); ivmatl.anohse@yandex.ru (I.S.M.); stropa99@mail.ru (P.D.S.); dan64rus@gmail.com (D.D.D.); goryachevaiy@mail.ru (I.Y.G.)

* Correspondence: olga.goryacheva.93@mail.ru

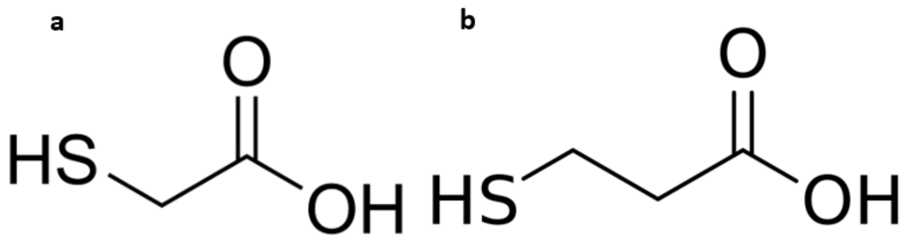

Figure S1. Formulas of (**a**) Thioglycolic acid and (**b**) 3-mercaptopropionic acid.

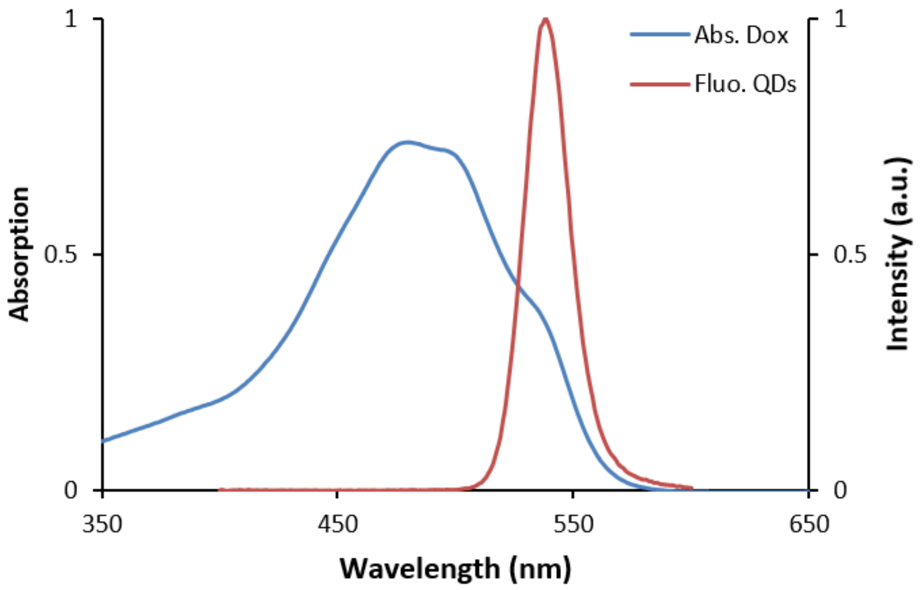

Figure S2. An overlap between the QDs fluorescence spectrum (**red**) and DOX absorption spectrum (**blue**).

Table S1. Correspondence of DOX concentration in μg/mL and μM (DOX Molecular Weight 543.5).

**μg/mL**	0	0.25	0.05	0.75	1	2.5	5	10	25	50	100	250	500
**μM**	0	0.46	0.92	1.38	1,8	4.6	9.2	18.4	45.6	92	184	460	920

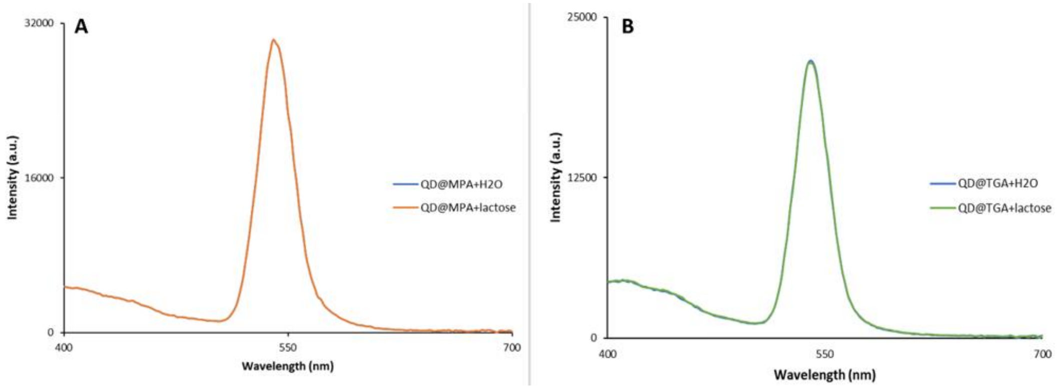

Figure S3. Fluorescence spectra of QDs stabilized with 3-mercaptopropionic acid (**A**) and thioglycolic acid (**B**) with and without lactose.

Two approaches were used for LOD calculations:

Formula S1

LOD = I_0 - 3σ

I_0 – QDs fluorescence intensity in the absence of DOX, middle for 11 measurements

σ - standard deviation of QDs fluorescence intensity in the absence of DOX

Because of in our case we have signal decreasing we have used minus in the formula

Formula S2

LOD = 3.3σ/S,

σ is the standard deviation of response

S is the slope of calibration curve ΔI vs C_{DOX}.

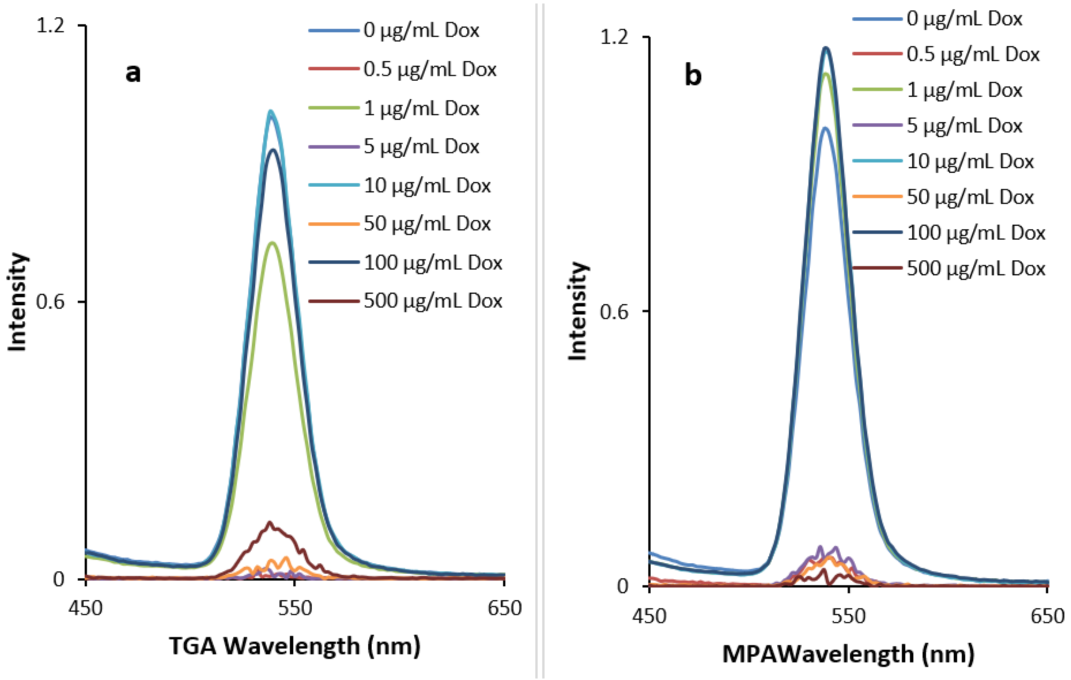

Figure S4. Fluorescence spectra of QDs stabilized with 3-mercaptopropionic acid (**a**) and thioglycolic acid (**b**) in X10 diluted plasma, spiked with DOX.
